# NGS identifies novel HLA-DQA1 and DPB1 associations with aplastic anemia in the Kazakhstani population

**DOI:** 10.3389/fimmu.2026.1752687

**Published:** 2026-02-05

**Authors:** Aida Turganbekova, Zhulduz Zhanzakova, Perizat Kanabekova, Dana Baimukasheva, Zhazira Saduakas, Didara Khamitova, Saniya Abdrakhmanova, Zhaksylyk Masalimov, Wassim Y. Almawi

**Affiliations:** 1 HLA Laboratory, Scientific and Production Center for Transfusiology, Astana, Kazakhstan; 2 Faculty of Natural Sciences, L.N. Gumilyov Eurasian National University, Astana, Kazakhstan; 3 School of Medicine, Nazarbayev University, Astana, Kazakhstan; 4 Faculty of Sciences, El-Manar University, Tunis, Tunisia

**Keywords:** aplastic anemia, DPB1 alleles, DQA1 alleles, HLA polymorphisms, next-generation sequencing

## Abstract

**Background:**

Aplastic anemia (AA) is a rare but serious blood disorder defined by autoimmune-driven destruction of bone marrow stem and progenitor cells. HLA polymorphisms are AA risk factors, with population-specific associations influencing disease susceptibility, treatment response, and transplant outcomes. While the genetic pathways driving AA development remain incompletely elucidated, a link between HLA variants and AA predisposition has been documented across diverse ethnic groups, though not in Central Asian communities, particularly in Kazakhstan.

**Objective:**

We investigated the relationship between HLA Class I and Class II alleles and the risk of AA in the Kazakhstani population using high-resolution NGS genotyping.

**Methods:**

The study included 91 patients with AA and 250 unrelated controls selected from the national registry of hematopoietic stem cell donors. HLA class I (A/C/B) and class II (DRB1/DQA1/DQB1/DPB1) high-resolution genotyping was conducted using NGS. Statistical significance was assessed with chi-square tests.

**Results:**

Class II alleles showed stronger associations with AA than Class I alleles. Novel HLA associations with strong effect sizes (ORs >69) were identified, including the first-ever reported associations between *HLA-DQA1* alleles and AA susceptibility. DRB1*05:05:01, DRB1*01:02:01, DQA1*05:05:01, DQA1*03:03:01, DQA1*03:02:01, DQA1*01:04:01, DQB1*02:02:01, DPB1*02:01:02, and DPB1*104:01:01 were associated with a higher AA risk in the Kazakhstani population. In contrast, DRB1*07:01:01, DRB1*15:01:01, DQA1*03:01:01, DQA1*01:01:01, DQA1*05:01:01, DQB1*02:01:01, and DPB1*02:01:01 were linked with reduced risk. Among Class I alleles, only B*40:02:01 showed a weak association with increased AA risk (*p* = 0.042), markedly lower than the strong Class II effects.

**Conclusions:**

Class II alleles, including those within *DQA1* and *DPB1*, are important genetic factors influencing AA susceptibility in Kazakhstan. It highlights the need for region-specific genetic profiling to improve disease risk assessment and guide therapeutic strategies.

## 1 Introduction

Aplastic anemia (AA) is a rare and potentially fatal blood disorder characterized by pancytopenia and decreased bone marrow cellularity, caused by the immune-mediated destruction of hematopoietic stem and progenitor cells (1). Mainly driven by autoreactive cytotoxic T cells and pro-inflammatory cytokines, including TNF-α and interferon-γ (1, 2), this results in bone marrow failure and a decreased production of adequate blood cells (3). The global incidence of AA varies by region, with estimates ranging from 2 to 6 individuals per million annually in Western countries, whereas East Asian populations report rates 2–3 times higher (4, 5). Risk factors for AA include a combination of environmental exposures, acquired factors, and genetic predispositions (2, 3). These encompass viral infections, autoimmune diseases, inherited bone marrow failure syndromes, as well as exposure to toxins, xenobiotics, and ionizing radiation (6, 7). Overall, this underscores the link between inherited predisposition and environmental triggers in the development of AA.

Located within the major histocompatibility complex (MHC) region on chromosome 6, the human leukocyte antigen (HLA) system plays a vital role in immune recognition and self-tolerance (8, 9). Structurally, HLA molecules are cell-surface glycoproteins composed of a heavy α-chain (and β2-microglobulin in Class I) or both α- and β-chains (for Class II), forming a peptide-binding groove that displays processed antigenic fragments to T cells (8, 10). HLA Class I molecules function in presenting endogenous peptides to CD8^+^ cytotoxic T cells, leading to the destruction of infected or malignant cells, while HLA Class II molecules present exogenous peptides to CD4^+^ helper T cells, triggering and regulating immune responses (11).

Typically, HLA typing is conducted using serology, PCR-based methods (SSP and SSO), and high-resolution next-generation sequencing (NGS), which provides the highest allelic accuracy (9). NGS-based typing enables the detection of allelic variants that were not routinely examined in earlier serological or low-resolution PCR-based studies, revealing novel disease associations. Specific HLA alleles and haplotypes are strongly associated with altered risks of autoimmune, infectious, and hematologic diseases (12, 13), including AA (14–16), due to their impact in shaping T-cell reactivity and immune tolerance.

Previous studies have identified significant interrelationships between specific Class I and Class II alleles and susceptibility to AA, with marked variation across ethnic groups. For example, DRB1*15:01 and DQB1*06:02 were linked with heightened susceptibility to AA, whereas DRB1*04:05 and DQB1*04:01 appear protective of AA in East Asians (17–19). In contrast, DRB1*03:01, DRB1*15, and DQB1*02:01 were associated with an altered AA risk in Europeans (20, 21). Furthermore, A*02:01, B*14:02, and DRB1*15:01 were marked with increased odds of severe AA in Egyptians (22). This underscores the distinct population-specific HLA risk profiles in AA, highlighting the need for ethnicity-tailored genetic studies to optimize therapeutic strategies across diverse ethnic populations (23).

Stretching across Central Asia and Eastern Europe, Kazakhstan is a transcontinental country with an area of 2,724,900 km2, and its population has been shaped by centuries of mixing Turkic, Mongolic, European, and Persian ancestries (9). Kazakhstan is home to over 120 ethnic groups, with ethnic Kazakhs making up 70%, Russians 15.1%, Uzbeks 3.2%, and other ethnicities, notably Ukrainians (1.9%) and Uighurs (1.5%) (9, 24). Despite this genetic diversity and the critical need for HLA matching in stem cell transplantation for AA treatment, HLA association studies in Kazakhstani populations are virtually nonexistent (25). This represents a significant gap in precision medicine in Central Asia, where population-specific genetic factors may strongly influence disease risk, treatment responses, and transplant outcomes (23, 25).

Using high-resolution NGS typing, this study aims to explore the connection between HLA Class I (*HLA-A*, *-B*, *-C*) and Class II (*HLA-DRB1*, *-DQA1*, *-DQB1*, *-DPB1*) alleles and susceptibility to AA in the Kazakh population. We aim to identify genetic markers that contribute to the pathogenesis of AA by establishing the first comprehensive HLA risk profile for AA in Central Asia and identifying novel allelic associations using high-resolution NGS that were not detectable in previous low-resolution studies. This will provide data to inform donor registry optimization and transplant matching strategies, laying the groundwork for future studies examining HLA-guided treatment personalization and outcome prediction in this genetically diverse population.

## 2 Methods

### 2.1 Study subjects

This retrospective study examined 91 patients with AA, with a mean age of 27 years (range: 3–71), who underwent HLA genotyping at the Research and Production Centre for Transfusiology in Astana, Kazakhstan, between December 2024 and May 2025. All participants self-identified as ethnic Kazakhs. HLA typing in AA patients was performed as part of routine pre-transplant evaluation. The diagnosis of AA was made following the criteria of the International Agranulocytosis and Aplastic Anemia Study Group (26). All patients had acquired, immune-mediated aplastic anemia, confirmed by standard clinical and marrow criteria which includes hypocellular bone marrow (typically less than 25% cellularity), along with two or more of the following: absolute neutrophil count below 0.5 × 10^9^/L, platelet count below 20 × 10^9^/L, and reticulocyte count below 20 × 10^9^/L, with no evidence of congenital marrow failure syndromes. The median time from diagnosis to enrollment was 3.2 months (Interquartile Range [IQR]: 1.0–5.5 months). Clinical outcome data, including treatment response, disease progression, and survival, were not collected as the study focused solely on identifying HLA associations with AA susceptibility.

Inclusion criteria consisted of normal karyotype, bone marrow biopsy for cellularity assessment, complete high-resolution *HLA-A*, *-B*, *-C*, -*DRB1*, *-DQA1*, *-DQB1*, and *-DPB1* typing by NGS, and informed consent. Exclusion criteria included ongoing hematological malignancies such as paroxysmal nocturnal hemoglobinuria and myelodysplastic syndrome, secondary AA caused by radiation, chemotherapy, viral hepatitis, or autoimmune diseases, incomplete or low-resolution HLA typing, unconfirmed AA diagnosis, and withdrawal of consent. Peripheral blood samples were collected at the time of initial diagnosis or before initiation of immunosuppressive therapy, and genotyping was performed concurrently with diagnostic hematologic and immunologic assessments.

The control group included 250 unrelated hematopoietic stem cell donors recruited from the National Hematopoietic Stem Cell Donor Registry of Kazakhstan, which reflects the broad tribal and sub-ethnic structure of the Kazakh population and provides a geographically and genetically representative reference cohort for HLA studies. All controls underwent comprehensive NGS-based HLA typing at three (Class I) and four (Class II) loci. The mean age of controls was 29.4 years (range: 18–62), with a male-to-female ratio of 1.1:1. These demographics were comparable to those of the patient group, minimizing confounding by age or sex-related HLA variation. Insofar as Kazakhstan is home to over 120 ethnic groups, both cases and controls were limited to self−identified ethnic Kazakhs and recruited from the same provinces (Astana, Akmola, Karaganda, Pavlodar, and East Kazakhstan). Cases and controls belonging to non-Kazakh ethnic groups, including Russians, Uzbeks, Uighurs, Koreans, and others, were excluded to minimize population stratification, ensure comparable ethnic composition and sub−regional ancestry between groups, and minimize confounding from inter−ethnic HLA frequency differences.

Allele frequencies of *HLA-A*, -*B*, -*C*, -*DRB1*, -*DQA1*, -*DQB1*, and -*DPB1* in the control group were compared with previously published HLA frequencies from ethnically matched Kazakh and Central Asian populations, including national bone marrow donor registries and population-based studies. This confirmed the representativeness of the control cohort and minimized potential sampling bias. Concordance between our control frequencies and reference datasets was evaluated qualitatively for major alleles and quantitatively for high-frequency variants. Exclusion criteria involved personal or family history of hematological disorders, autoimmune or chronic inflammatory conditions, and unclear ethnicity. The study protocol was approved by the Local Commission on Bioethics of the Scientific and Production Center for Transfusiology (SPCT/2024/6). It was conducted in accordance with the principles outlined in the Declaration of Helsinki.

### 2.2 DNA extraction and HLA typing

HLA typing for both AA patients and healthy control donors was performed at the same certified facility: the Scientific and Production Center for Transfusiology (Astana, Kazakhstan), using an identical workflow and instrumentation. Both groups were processed with the same sequencing platform (MiSeq), library preparation method, and HLA Twin™ v4.9.0 bioinformatics pipeline to ensure consistency and to minimize technical bias. Peripheral blood samples were collected in EDTA-K_2_ tubes, and genomic DNA was extracted using the automated BEX12 system (Inno-Train Diagnostics, Germany) via magnetic particle-based separation. DNA was quantified with a Qubit™ fluorometer (Thermo Fisher Scientific, Almaty, Kazakhstan) and adjusted to 36 ng/μL. High-resolution HLA genotyping was performed using the Holotype HLA 24/7 kit (Omixon Inc., Hungary) with next-generation sequencing (NGS) for *HLA-A*, *-B*, -*C*, -*DRB1*, *-DQB1*, *-DQA1*, and *-DPB1*. Whole coding regions of the tested HLA loci were amplified, including the 5′ and 3′ untranslated regions. *HLA-DRB1* amplification targeted introns 1-4, while *HLA-DPB1* included the UTR and exonic regions from introns 1-3.

Amplification success was confirmed by 2% agarose gel electrophoresis. Amplicon concentrations were measured using QuantiFluor^®^ dsDNA System (Promega, USA). Following purification, fragmentation, end-repair, and adapter ligation, the prepared library (600 μL, 9 pM) was sequenced on the MiSeq platform (Illumina Inc., USA). Bioinformatic analysis was done using HLA Twin™ software v4.9.0 (Omixon Inc., Hungary) at a six-digit resolution to reflect the full allelic resolution achieved through NGS typing, with only complete, validated profiles included.

### 2.3 Statistical analysis

HLA allele distributions were determined through direct gene counting. Comparisons of HLA allele frequencies between patients and controls were analyzed using Chi-square tests, with Yates’ correction for continuity applied when the expected cell counts were less than 5. Odds ratios (ORs) and 95% confidence intervals (CIs) were used to assess the strength of association between specific HLA alleles and the risk of AA. Fisher’s exact test was applied when allele counts were fewer than five or absent in one group. Genotype frequencies were calculated for each HLA locus in the control group. To exclude cryptic population stratification (Wahlund effect), Hardy–Weinberg equilibrium (HWE) was tested for all HLA loci in the control cohort using a Monte Carlo exact test for multi-allelic loci (10,000 replicates) based on observed allele frequencies, which is appropriate for highly polymorphic HLA loci. Conformity of all Class II loci to HWE further confirms the absence of residual ethnic admixture or Wahlund effects within the ethnic-Kazakh control cohort; p< 0.05 was considered evidence of deviation.

Adjusted standardized residuals (ASR; Haberman residuals) were used for quantifying the contribution of individual alleles to the overall χ² and is robust for polymorphic HLA data with sparse or zero-count cells. and were calculated as per: ASR = (O − E)/√(E × (1 − row proportion) × (1 − column proportion)), where O and E are observed and expected counts. Alleles with |ASR| ≥ 1.96 were considered statistically significant contributors to the association signal. Allele- and haplotype-specific p-values and odds ratios (ORs) were first calculated as unadjusted estimates, followed by age- and sex-adjusted ORs were computed using multivariable logistic regression to account for potential confounding by demographic variables that influence AA risk. While neither age nor sex was significantly associated with individual HLA allele frequencies (all p > 0.10), both were retained in the adjusted model for completeness and comparability with previous HLA association studies.

A Bonferroni correction was applied to the age- and sex-adjusted p-values from logistic regression to control for multiple testing. These corrected adjusted p-values are reported as “Corrected p” in all tables. Because AA is a rare disease, and HLA loci are highly polymorphic, some alleles were absent in one group, leading to zero-frequency cells. These were handled using Fisher’s exact test and Haberman ASR, which are robust for sparse contingency tables. To exclude technical or population-genetic artifacts, all Class II loci in controls were tested for HWE using Monte-Carlo exact tests, confirming the absence of genotyping errors, allele dropout, or hidden substructure. Rather than sampling bias, zero-frequency alleles reflect true population-restricted variants, and their large ORs indicate enrichment among cases.

Linkage disequilibrium (LD) analysis was performed using the expectation–maximization (EM) algorithm implemented in a custom R pipeline to detect non-random associations between alleles and to estimate haplotype frequencies from unphased genotypes. Pairwise LD strength was summarized as the average absolute D′ and r² values across allelic combinations and visualized as heatmaps depicting multi-locus LD structure separately in patients and controls. The frequencies of three-locus (Class I) and four-locus (Class II) haplotypes were estimated using the expectation-maximization algorithm based on maximum-likelihood principles, implemented in Arlequin version 3.5.2.2. All statistical analyses were carried out in R (version 4.3.0) with a predefined significance level of 0.05. For consistency, p-values were reported in 3-digit decimal notation throughout the tables and text, and values<0.001 are shown as “<.001”.

## 3 Results

### 3.1 HLA class I allele distribution

*HLA-A*, *HLA-C*, and *HLA-B* class I allele distributions were examined in 91 patients with AA and 250 controls. In the control group (n = 250), genotype distributions were in Hardy–Weinberg equilibrium for all loci except HLA−A, which showed a mild deviation (p = 0.034). HLA−C, −B, −DRB1, −DQA1, −DQB1, and −DPB1 all conformed to HWE, indicating no evidence of genotyping error, allele dropout, or population substructure. The alignment of DRB1, DQA1, DQB1, and DPB1 with HWE further supports the representativeness of the control cohort. Bonferroni−corrected, age− and sex−adjusted p−values are reported in **Tables 1**-**7**, and full control genotype frequencies appear in **Supplementary Table S1**. Overall, the control allele distributions closely matched those previously reported for ethnic Kazakh populations, suggesting minimal sampling bias and reinforcing the validity of the case–control comparisons.

**Table 1:** HLA-A* allele distribution among aplastic anemia cases and controls.

A-Allele	Patients *^1^*	Controls *^1^*	Chi square	*p ^2^*	*p*_adj_^3^	OR_adj_ (95% CI) ^3^	Corrected *p ^4^*
A*33:01:01	0.01648	0.00714	1.402	.391	.236	2.330 (0.552, 8.040)	.992
A*26:01:01	0.06044	0.05000	0.318	.698	.573	1.222 (0.608, 2.456)	1.000
A*29:01:01	0.01099	0.01571	0.222	.738	.637	0.696 (0.153, 3.168)	1.000
A*32:01:01	0.02198	0.02714	0.152	.792	.697	0.805 (0.271, 2.397)	1.000
A*30:01:01	0.02198	0.01857	0.089	.754	.766	1.188 (0.383, 3.686)	1.000
A*01:01:01	0.07692	0.08571	0.145	.757	.703	0.889 (0.485, 1.629)	1.000
A*68:01:02	0.01648	0.03429	1.543	.309	.214	0.472 (0.141, 1.585)	.987
A*31:01:02	0.04396	0.04714	0.033	.823	.856	0.929 (0.422, 2.048)	1.000
A*24:02:01	0.22527	0.18857	1.234	.328	.267	1.251 (0.842, 1.859)	.996
A*02:01:01	0.20330	0.17714	0.662	.450	.416	1.185 (0.787, 1.786)	1.000
A*02:05:01	0.01648	0.01000	0.542	.689	.462	1.659 (0.425, 6.481)	1.000
A*02:06:01	0.06593	0.03429	3.695	.068	.055	1.988 (0.975, 4.057)	.639
A*02:07:01	0.01099	0.02857	1.836	.259	.175	0.378 (0.087, 1.631)	.969
A*33:03:01	0.03846	0.05286	0.632	.549	.427	0.717 (0.314, 1.635)	1.000
A*03:01:01	0.05495	0.06286	0.157	.729	.692	0.867 (0.428, 1.758)	1.000
A*03:02:01	0.01648	0.00429	3.181	.121	.075	3.894 (0.779, 19.456)	.754
A*11:01:01	0.04945	0.08857	2.987	.096	.084	0.535 (0.261, 1.099)	.794
A*23:01:01	0.01648	0.01857	0.035	1.000	.851	0.886 (0.250, 3.142)	1.000

1. Allele frequency).

2. Crude (unadjusted) analysis.

3. Adjusted for age and gender.

4. Denotes the Bonferroni-adjusted age- and sex-adjusted p-value derived from logistic regression, calculated as: $p_{c}=1-{\left(1-p_{adj}\right)}^{n}$, where n  is the number of alleles tested at that locus.

Among the 18 different *HLA-A* alleles identified, A*02:01:01 (20.33% in patients, 17.71% in controls) and A*24:02:01 (22.53% in patients, 18.86% in controls) were the most common alleles (**Table 1**). Overall, no statistically significant associations were found between any *HLA-A* alleles and AA risk even before applying the Bonferroni correction for multiple comparisons (**Table 1**).

Of the 16 *HLA-C* alleles analyzed, C*07:02:01 was the most common in patients and controls (15.4%, 10.3%). After adjusting for age and gender, C*07:06:01 (*p* = .007), C*08:01:01 (*p* = .030) showed positive associations, while C*04:01:01 (*p* = .034) was negatively associated with AA risk before correction (**Table 2**). However, none remained significant after Bonferroni correction for multiple testing (all corrected *p* >.050) (**Table 2**).

**Table 2:** HLA-C* allele distribution among aplastic anemia cases and controls.

C-Allele	Patients ^1^	Controls ^1^	Chi square	*P ^2^*	*p*_adj_^2^	OR_adj_ (95% CI) ^2^	Corrected *p*^3^
C*07:06:01	0.01099	0.00000	7.193	.070	.007	NA	.137
C*08:01:01	0.04396	0.01714	4.686	.09	.030	2.636 (1.061, 6.548)	.473
C*04:01:01	0.05495	0.10714	4.519	.036	.034	0.484 (0.245, 0.957)	.516
C*07:02:01	0.15385	0.10286	3.736	.061	.053	1.586 (0.990, 2.539)	.681
C*03:04:01	0.12637	0.08286	3.280	.101	.070	1.601 (0.958, 2.675)	.782
C*07:04:01	0.01099	0.03429	2.740	.121	.098	0.313 (0.073, 1.337)	.885
C*15:02:01	0.02747	0.05714	2.626	.115	.105	0.466 (0.181, 1.198)	.903
C*12:03:01	0.03846	0.07143	2.597	.111	.107	0.520 (0.232, 1.167)	.907
C*16:04:01	0.01099	0.00286	2.116	.175	.146	3.878 (0.542, 27.718)	.964
C*05:01:01	0.03846	0.02286	1.385	.279	.239	1.710 (0.693, 4.221)	.997
C*02:02:02	0.01648	0.03143	1.171	.312	.279	0.517 (0.153, 1.745)	.999
C*03:03:01	0.05495	0.03714	1.169	.332	.280	1.507 (0.713, 3.185)	.999
C*12:02:02	0.01099	0.00429	1.151	.578	.283	2.581 (0.428, 15.566)	.999
C*08:02:01	0.02747	0.01571	1.121	.348	.290	1.769 (0.607, 5.158)	.999
C*06:02:01	0.10440	0.12429	0.540	.484	.462	0.821 0.486, 1.389)	1.000
C*08:03:01	0.00549	0.01143	0.504	.682	.478	0.478 (0.059, 3.846)	1.000
C*14:02:01	0.02198	0.02571	0.083	1.000	.773	0.851 (0.285, 2.547)	1.000
C*12:02:01	0.02747	0.03143	0.076	.764	.783	0.871 (0.325, 2.331)	1.000
C*01:02:01	0.07143	0.07286	0.004	1.000	.947	0.979 (0.520, 1.842)	1.000
C*07:01:01	0.05495	0.05429	0.001	1.000	.972	1.013 (0.495, 2.074)	1.000
C*03:02:02	0.05495	0.05429	0.001	1.000	.972	1.013 (0.495, 2.074)	1.000

1. Allele frequency).

2. Crude (unadjusted) analysis.

3. Adjusted for age and gender.

4. Denotes the Bonferroni-adjusted age- and sex-adjusted p-value derived from logistic regression, calculated as: $p_{c}=1-{\left(1-p_{adj}\right)}^{n}$, where n is the number of alleles tested at that locus.

The greatest diversity was seen at the *HLA-B* locus, with 31 distinct alleles identified. B*40:02:01 (11.5%) and B*07:02:01 (9.3%) were the most prevalent alleles among patients (**Table 3**). While the frequency of B*40:02:01 demonstrated the strongest association with increased AA *risk* (*p = .*002), this was borderline after Bonferroni correction (corrected *p = .*060) (**Table 3**). B*44:03:02 (*p* = .007), B*67:01:01 (*p = .*007), and B*73:01:01 (*p* = .048) showed nominal significance but wide confidence intervals. No alleles achieved statistical significance after correction for multiple comparisons (**Table 3**).

**Table 3:** HLA-B* allele distribution among aplastic anemia cases and controls.

B-Allele	Patients ^1^	Controls ^1^	Chi square	*p ^2^*	*p*_adj_^3^	OR_adj_ (95% CI) ^3^	Corrected *p ^4^*
B*40:02:01	0.11538	0.05286	9.192	.007	.002	2.337 (1.332, 4.102)	.060
B*44:03:02	0.01099	0.00000	7.193	.122	.007	19.404 (0.927, 405.989)	.196
B*67:01:01	0.01099	0.00000	7.193	.122	.007	19.404 (0.927, 405.989)	.196
B*73:01:01	0.01099	0.00143	3.895	.360	.048	7.767 (0.700, 86.136)	.782
B*07:02:01	0.09341	0.05857	2.853	.145	.091	1.656 (0.917, 2.989)	.948
B*54:01:01	0.03297	0.01429	2.830	.199	.093	2.352 (0.843, 6.560)	.951
B*49:01:01	0.03297	0.01429	2.830	.199	.093	2.352 (0.843, 6.560)	.951
B*44:03:01	0.00549	0.02143	2.059	.262	.151	0.252 (0.033, 19.23)	.994
B*44:02:01	0.04396	0.02429	2.029	.267	.154	1.847 (0.784, 4.351)	.994
B*35:01:01	0.02747	0.05143	1.870	.249	.171	0.521 (0.202, 1.347)	.997
B*40:01:01	0.00000	0.01429	1.771	.240	.183	0.180 (0.011, 3.089)	.998
B*38:01:01	0.01099	0.02714	1.622	.311	.203	0.398 (0.092, 1.726)	.999
B*27:05:02	0.01099	0.02714	1.622	.311	.203	0.398 (0.092, 1.726)	.999
B*46:01:01	0.01099	0.02571	1.413	.375	.235	0.421 (0.097, 1.831)	1.000
B*18:01:01	0.01648	0.03143	1.171	.409	.279	0.517 (0.153, 1.745)	1.000
B*14:02:01	0.02747	0.01571	1.121	.514	.290	1.769 (0.607, 5.158)	1.000
B*15:18:01	0.01099	0.02143	0.833	.539	.361	0.507 (0.115, 2.239)	1.000
B*35:03:01	0.03297	0.04571	0.569	.595	.451	0.712 (0.293, 1.729)	1.000
B*37:01:01	0.01648	0.01000	0.542	.769	.462	1.659 (0.425, 6.481)	1.000
B*13:02:01	0.05495	0.07000	0.524	.599	.469	0.772 (0.383, 1.556)	1.000
B*48:01:01	0.03846	0.02857	0.476	.653	.490	1.360 (0.566, 3.268)	1.000
B*15:01:01	0.02198	0.03143	0.451	.667	.502	0.693 (0.236, 2.035)	1.000
B*52:01:01	0.02747	0.03571	0.299	.760	.585	0.763 (0.288, 2.021)	1.000
B*35:02:01	0.01099	0.00714	0.271	1.000	.602	1.544 (0.297, 8.026)	1.000
B*57:01:01	0.01099	0.01571	0.222	.903	.637	0.696 (0.153, 3.168)	1.000
B*39:01:01L	0.01099	0.01571	0.222	.903	.637	0.696 (0.153, 3.168)	1.000
B*50:01:01	0.02198	0.02714	0.152	.870	.697	0.805 (0.271, 2.397)	1.000
B*51:01:01	0.07143	0.08000	0.147	.835	.701	0.885 (0.473, 1.656)	1.000
B*40:06:01	0.01099	0.01429	0.117	1.000	.732	0.767 (0.167, 3.530)	1.000
B*08:01:01	0.03297	0.03714	0.072	.937	.788	0.884 (0.358, 2.180)	1.000
B*58:01:01	0.05495	0.06000	0.067	.949	.796	0.911 (0.448, 1.852)	1.000

1. Allele frequency).

2. Crude (unadjusted) analysis.

3. Adjusted for age and gender.

4. Denotes the Bonferroni-adjusted age- and sex-adjusted p-value derived from logistic regression, calculated as: $p_{c}=1-{\left(1-p_{adj}\right)}^{n}$, where n is the number of alleles tested at that locus.

### 3.2 HLA class II allele distribution

Of the 25 *HLA-DRB1* alleles identified, the most prevalent in patients were DRB1*01:02:01 (18.3%) and DRB1*05:05:01 (15.6%). After adjustments for age, gender, and multiple comparisons, several HLA-DRB1* alleles were significantly associated with a higher risk of AA. These comprised DRB1*05:05:01, DRB1*01:02:01, DRB1*03:03:01, DRB1*05:01:01, DRB1*02:01:01, DRB1*01:03:01, DRB1*03:02:01, and DRB1*01:04:01 with adjusted odds ratios exceeding 25 and corrected *p* <.0001, as well as DRB1*06:01:01 (*p* = .020), and DRB1*05:03:01 (corrected *p* = .020). On the other hand, alleles like DRB1*07:01:01 and DRB1*15:01:01 (both with corrected *p* <.001), and DRB1*04:01:01 (Corrected *p* = .027) and DRB1*05:03:01 (Corrected *p* = .045) were more frequent in controls, suggesting protective associations (**Table 4**).

**Table 4:** HLA-DRB1* allele distribution among aplastic anemia cases and controls.

DRB1* Allele	Patients ^1^	Controls ^1^	Chi square	*p ^2^*	*p*_adj_^3^	OR_adj_ (95% CI) ^3^	Corrected *p*^4^	ASR
DRB1*05:05:01	**0.15556**	**0.00000**	**111.149**	**<.0001**	**<.0001**	**261.826 (15.897, 4312.308)**	**<.0001**	**+10.7**
DRB1*01:02:01	**0.18333**	**0.00857**	**103.249**	**<.0001**	**<.0001**	**25.966 (10.686, 63.097)**	**<.0001**	**+9.8**
DRB1*03:03:01	**0.11111**	**0.00000**	**78.398**	**<.0001**	**<.0001**	**178.944 (10.766, 2974.315)**	**<.0001**	**+8.6**
DRB1*05:01:01	**0.11111**	**0.00000**	**78.398**	**<.0001**	**<.0001**	**178.944 (10.766, 2974.315)**	**<.0001**	**+8.6**
DRB1*02:01:01	**0.10556**	**0.00000**	**74.348**	**<.0001**	**<.0001**	**169.161 (10.160, 2816.443)**	**<.0001**	**+8.3**
DRB1*01:03:01	**0.08333**	**0.00000**	**58.246**	**<.0001**	**<.0001**	**131.211 (7.811, 2204.182)**	**<.0001**	**+7.3**
DRB1*03:02:01	**0.05556**	**0.00000**	**38.347**	**<.0001**	**<.0001**	**86.279 (5.031, 1479.711)**	**<.0001**	**+5.9**
DRB1*01:04:01	**0.04444**	**0.00000**	**30.465**	**<.0001**	**<.0001**	**69.035 (3.965, 1201.938)**	**<.0001**	**+5.3**
DRB1*07:01:01	**0.00000**	**0.12714**	**24.497**	**<.0001**	**<.0001**	**0.019 (0.001, 0.306)**	**.0002**	**-6.9**
DRB1*15:01:01	**0.00000**	**0.09571**	**17.686**	**<.0001**	**<.0001**	**0.026 (0.002, 0.422)**	**.0006**	**-6.1**
DRB1*06:01:01	**0.01667**	**0.00000**	**11.052**	**.026**	**.001**	**27.625 (1.420, 537.296)**	**.020**	**-4.0**
DRB1*05:03:01	**0.01667**	**0.00000**	**11.052**	.026	**.001**	**27.625 (1.420, 537.296)**	**.020**	+2.7
DRB1*04:01:01	**0.01667**	**0.08714**	**10.546**	**.002**	**.001**	**0.178 (0.055, 0.573)**	**.027**	**-4.0**
DRB1*13:01:01	**0.00000**	**0.05571**	**9.541**	**.002**	**.002**	**0.046 (0.003, 0.758)**	**.045**	**-4.7**
DRB1*14:01:01	0.00000	0.04429	7.318	.008	.007	0.059 (0.004, 0.967)	.146	**-4.1**
DRB1*13:01:01	0.00000	0.05571	9.541	.002	.002	0.046 (0.003, 0.758)	.149	-4.7
DRB1*14:01:01	0.00000	0.04429	7.318	.008	.007	0.059 (0.004, 0.967)	.168	**-4.1**
DRB1*01:05:01	0.01111	0.00000	7.282	.122	.007	19.622 0.938, 410.554)	.178	+2.8
DRB1*11:01:01	0.00000	0.04286	7.043	.011	.008	0.061 (0.004, 1.001)	.289	-2.8
DRB1*03:01:01	0.03889	0.10143	6.933	.013	.008	0.358 0.162, 0.793)	.372	-2.8
DRB1*13:02:01	0.00000	0.03714	5.952	.016	.015	0.071 (0.004, 1.163)	.419	-2.6
DRB1*12:01:01	0.00000	0.03429	5.412	.025	.020	0.076 (0.005, 1.264)	.525	-2.5
DRB1*09:01:02	0.00000	0.03286	5.142	.031	.023	0.080 (0.005, 1.321)	.680	-2.5
DRB1*15:02:01	0.00000	0.03000	4.607	.039	.032	0.088 (0.005, 1.452)	.527	-2.4
DRB1*01:01:01	0.03333	0.07429	3.900	.076	.048	0.430 (0.182, 1.017)	.677	-2.2

ASR, Adjusted Standardized Residuals; Boldface indicates statistically significant differences.

1. Allele frequency).

2. Crude (unadjusted) analysis.

3. Adjusted for age and gender.

4. Denotes the Bonferroni-adjusted age- and sex-adjusted p-value derived from logistic regression, calculated as: $p_{c}=1-{\left(1-p_{adj}\right)}^{n}$, where n is the number of alleles tested at that locus.

The most notable associations were seen with the *HLA-DQA1* locus. DQA1*05:05:01, DQA1*03:03:01, DQA1*03:02:01, and DQA1*01:04:01 were dramatically overrepresented in patients (Corrected *p* <.0001 for all four *DQA1* alleles). These were exclusively found in patients and were significantly associated with an increased risk of AA, with adjusted odds ratios exceeding 69 (**Table 5**). In addition, significant negative links with AA were found for DQA1*03:01:01 (*p* <.0001), DQA1*01:01:01 (*p* = .002), and DQA1*05:01:01 (p = .0013), which were under-represented in patients, and linked with reduced risk of AA, with adjusted OR ranging from 0.440-0.150 (**Table 5**).

**Table 5:** HLA-DQA1* allele distribution among aplastic anemia cases and controls.

DQA1* Allele	Patients ^1^	Controls ^1^	Chi square	*p ^2^*	*p*_adj_^3^	OR_adj_ (95% CI) ^3^	Corrected *p*^3^	ASR
DQA1*05:05:01	**0.15556**	**0.00000**	**111.149**	**<.0001**	**<.0001**	**261.826 (15.897, 4312.308)**	**<.0001**	**+9.48**
DQA1*03:03:01	**0.11111**	**0.00000**	**78.398**	**<.0001**	**<.0001**	**178.944 (10.766, 2974.315)**	**<.0001**	**+7.98**
DQA1*03:02:01	**0.05556**	**0.00000**	**38.347**	**<.0001**	**<.0001**	**86.279 (5.031, 1479.711)**	**<.0001**	**+5.57**
DQA1*01:04:01	**0.04444**	**0.00000**	**30.465**	**<.0001**	**<.0001**	**69.035 (3.965, 1201.938)**	**<.0001**	**+4.86**
DQA1*03:01:01	**0.03889**	**0.20857**	**28.701**	**<.0001**	**<.0001**	**0.154 (0.071, 0.334)**	**<.0001**	**-7.32**
DQA1*01:01:01	**0.03333**	**0.15714**	**19.178**	**<.0001**	**<.0001**	**0.185 (0.080, 0.428)**	**.0002**	**-5.86**
DQA1*05:01:01	**0.11111**	**0.22143**	**10.937**	**.002**	**.001**	**0.440 (0.267, 0.723)**	**.0013**	**-3.42**
DQA1*01:05:01	0.01111	0.00000	7.282	.122	.007	19.622 (0.938, 410.558)	.093	+1.67
DQA1*01:01:02	0.00556	0.00000	3.648	.598	.056	11.708 (0.475, 288.615)	.555	+1.14
DQA1*01:02:01	0.18333	0.13714	2.442	.200	.118	1.412 (0.915, 2.181)	.828	+1.27
DQA1*01:03:01	0.08333	0.11143	1.196	.328	.274	0.725 (0.406, 1.293)	.989	-0.91
DQA1*02:01:01	0.10556	0.12714	0.620	.483	.431	0.810 (0.479, 1.369)	1.000	-0.61
DQA1*04:01:01	0.01667	0.01000	0.566	.769	.452	1.678 (0.430, 6.554)	1.000	+0.55
DQA1*06:01:01	0.01667	0.02571	0.503	.660	.478	0.642 (0.187, 2.205)	1.000	-0.45

ASR, Adjusted Standardized Residuals; Boldface indicates statistically significant differences.

1. Allele frequency).

2. Crude (unadjusted) analysis.

3. Adjusted for age and gender.

4. Denotes the Bonferroni-adjusted age- and sex-adjusted p-value derived from logistic regression, calculated as: $p_{c}=1-{\left(1-p_{adj}\right)}^{n}$, where n is the number of alleles tested at that locus.

A total of 15 alleles were identified at the *HLA-DQB1* locus. Among these, DQB1*02:01:01 (21.1%) and DQB1*03:01:01 (21.7%) were the most common alleles in controls (**Table 6**). DQB1*02:02:01 showed a strong association with AA and was exclusively observed in patients (OR = 138.838), which remained significant after Bonferroni correction (*p* <.001). On the other hand, DQB1*02:01:01 was more prevalent in controls (21.1%) than in patients (11.5%) and was considered protective of AA (OR = 0.486). This remained significant after Bonferroni correction (corrected *p* = .049). The initial positive association of DQB1*06:02:01 (*p* = .006) and negative association of DQB1*05:01:01 (*p* = .026) with AA was lost significance after correction (**Table 6**).

**Table 6:** HLA-DQB1* allele distribution among aplastic anemia cases and controls.

DQB1* Allele	Patients ^1^	Controls ^1^	Chi square	*p ^2^*	*p*_adj_^3^	OR_adj_ (95% CI) ^3^	Corrected *p*^3^	ASR
DQB1*02:02:01	**0.08791**	**0.00000**	**61.563**	**<.001**	**<.001**	**138.838 (8.287, 2326.034)**	**<.001**	**+8.12**
DQB1*02:01:01	**0.11538**	**0.21143**	**8.602**	**.006**	**.003**	**0.486 (0.298, 0.794)**	**.049**	**-3.71**
DQB1*06:02:01	0.14835	0.08143	7.508	.016	.006	1.965 (1.203, 3.209)	.088	+2.66
DQB1*05:01:01	0.04396	0.09571	4.974	.042	.026	0.434 (0.205, 0.922)	.324	-2.23
DQB1*04:01:01	0.03297	0.01429	2.830	.199	.093	2.352 (0.843, 6.560)	.767	+1.38
DQB1*05:03:01	0.01648	0.04000	2.356	.206	.125	0.402 (0.121, 1.338)	.865	-1.46
DQB1*06:01:01	0.03297	0.06143	2.230	.197	.135	0.521 (0.218, 1.244)	.887	-1.27
DQB1*03:02:01	0.05495	0.08000	1.310	.345	.252	0.669 (0.334, 1.338)	.987	-1.05
DQB1*03:03:02	0.06593	0.04571	1.246	.397	.264	1.474 (0.743, 2.922)	.990	+0.88
DQB1*03:01:01	0.25275	0.21714	1.052	.393	.305	1.219 (0.834, 1.782)	.996	+0.74
DQB1*05:02:01	0.04396	0.03143	0.690	.607	.406	1.417 (0.620, 3.237)	1.000	+0.61
DQB1*06:04:01	0.01648	0.02571	0.530	.660	.467	0.635 (0.185, 2.180)	1.000	-0.52
DQB1*06:03:01	0.04945	0.05571	0.110	.886	.740	0.882 (0.419, 1.855)	1.000	-0.32
DQB1*04:02:01	0.01648	0.01429	0.048	1.000	.826	1.156 (0.315, 4.246)	1.000	+0.21
DQB1*06:09:01	0.01099	0.01143	0.002	1.000	.960	0.961 (0.202, 4.565)	1.000	-0.04

ASR, Adjusted Standardized Residuals; Boldface indicates statistically significant differences.

1. Allele frequency).

2. Crude (unadjusted) analysis.

3. Adjusted for age and gender.

4. Denotes the Bonferroni-adjusted age- and sex-adjusted p-value derived from logistic regression, calculated as: $p_{c}=1-{\left(1-p_{adj}\right)}^{n}$, where n is the number of alleles tested at that locus.

Highly significant differences were seen between patients and controls in the distribution of *HLA-DPB1* alleles. Of the 14 common *DPB1* alleles identified, DPB1*04:01:01 (32.7%) and DPB1*02:01:01 (17.7%) were the most common alleles in healthy controls (**Table 7**). Notably, DPB1*02:01:02 (7.1% vs. 0%, p<.0001) and DPB1*104:01:01 (3.3% vs. 0.0%, *p<*.0001) were found exclusively in AA cases and linked with high risk of AA (OR = 51.595, OR = 111.584) (**Table 7**). On the other hand, DPB1*02:01:01 was under-represented in AA patients (7.7%) compared with controls (17.7%) and thus was protective in AA patients (**Table 7**). The initial positive association of DPB1*05:01:01 (*p* = .025) with AA was lost significance after correction for multiple comparisons (**Table 7**).

**Table 7:** HLA-DPB1* allele distribution among aplastic anemia cases and controls.

DPB1* Allele	Patients ^1^	Controls ^1^	Chi square	*p ^2^*	*p*_adj_^3^	OR_adj_ (95% CI) ^3^	Corrected *p*^4^	ASR
DPB1*02:01:02	**0.07143**	**0.00000**	**49.694**	**<.0001**	**<.0001**	**111.584 (6.600, 1886.508)**	**<.0001**	**+7.38**
DPB1*104:01:01	**0.03297**	**0.00000**	**22.378**	**<.0001**	**<.0001**	**51.595 (2.893, 920.253)**	**<.0001**	**+5.02**
DPB1*02:01:01	**0.07692**	**0.17714**	**10.992**	.002	**.001**	**0.387 (0.217, 0.690)**	**.013**	**-3.82**
DPB1*05:01:01	0.12637	0.07429	5.037	.047	.025	1.803 (1.071, 3.034)	.297	+2.24
DPB1*04:01:01	0.40110	0.32714	3.509	.093	.061	1.377 (0.985, 1.927)	.586	+1.47
DPB1*03:01:01	0.06044	0.09143	1.783	.246	.182	0.639 (0.330, 1.239)	.940	-1.18
DPB1*04:02:01	0.09341	0.12857	1.676	.271	.196	0.698 (0.404, 1.206)	.952	-1.01
DPB1*17:01:01	0.02198	0.04000	1.342	.371	.247	0.539 (0.187, 1.558)	.981	-0.78
DPB1*02:02:01	0.00549	0.01571	1.124	.494	.289	0.346 (0.044, 2.698)	.992	-0.64
DPB1*14:01:01	0.01099	0.01857	0.497	.765	.481	0.587 (0.131, 2.626)	1.000	-0.43
DPB1*36:01:01	0.00000	0.00571	0.351	.694	.554	0.424 (0.023, 7.912)	1.000	-0.38
DPB1*13:01:01	0.02198	0.03000	0.338	.764	.561	0.727 (0.246, 2.144)	1.000	-0.31
DPB1*09:01:01	0.02198	0.02286	0.005	1.000	.943	0.961 (0.317, 2.909)	1.000	-0.05
DPB1*15:01:01	0.01099	0.01143	0.002	1.000	.960	0.961 (0.202, 4.565)	1.000	-0.02

ASR, Adjusted Standardized Residuals; Boldface indicates statistically significant differences.

1. Allele frequency).

2. Crude (unadjusted) analysis.

3. Adjusted for age and gender.

4. Denotes the Bonferroni-adjusted age- and sex-adjusted p-value derived from logistic regression, calculated as: $p_{c}=1-{\left(1-p_{adj}\right)}^{n}$, where n is the number of alleles tested at that locus.

ASRs confirmed that the strongest associations were driven by a limited number of Class II alleles. DQA1*05:05:01, DQA1*03:03:01, DQA1*03:02:01, DQB1*02:02:01, DPB1*02:01:02, and DPB1*104:01:01 showed very large positive ASRs (exceeding 1.96), indicating extreme over-representation among AA patients relative to controls. In contrast, protective alleles including DQA1*03:01:01, DQA1*01:01:01, DQB1*02:01:01, and DPB1*02:01:01 presented with large negative ASRs, consistent with depletion among cases. Collectively, this confirms that the extreme ORs arise from statistically dominant allelic effects rather than sparse-table artifacts.

### 3.3 Haplotype frequency distribution

**Figure 1:**
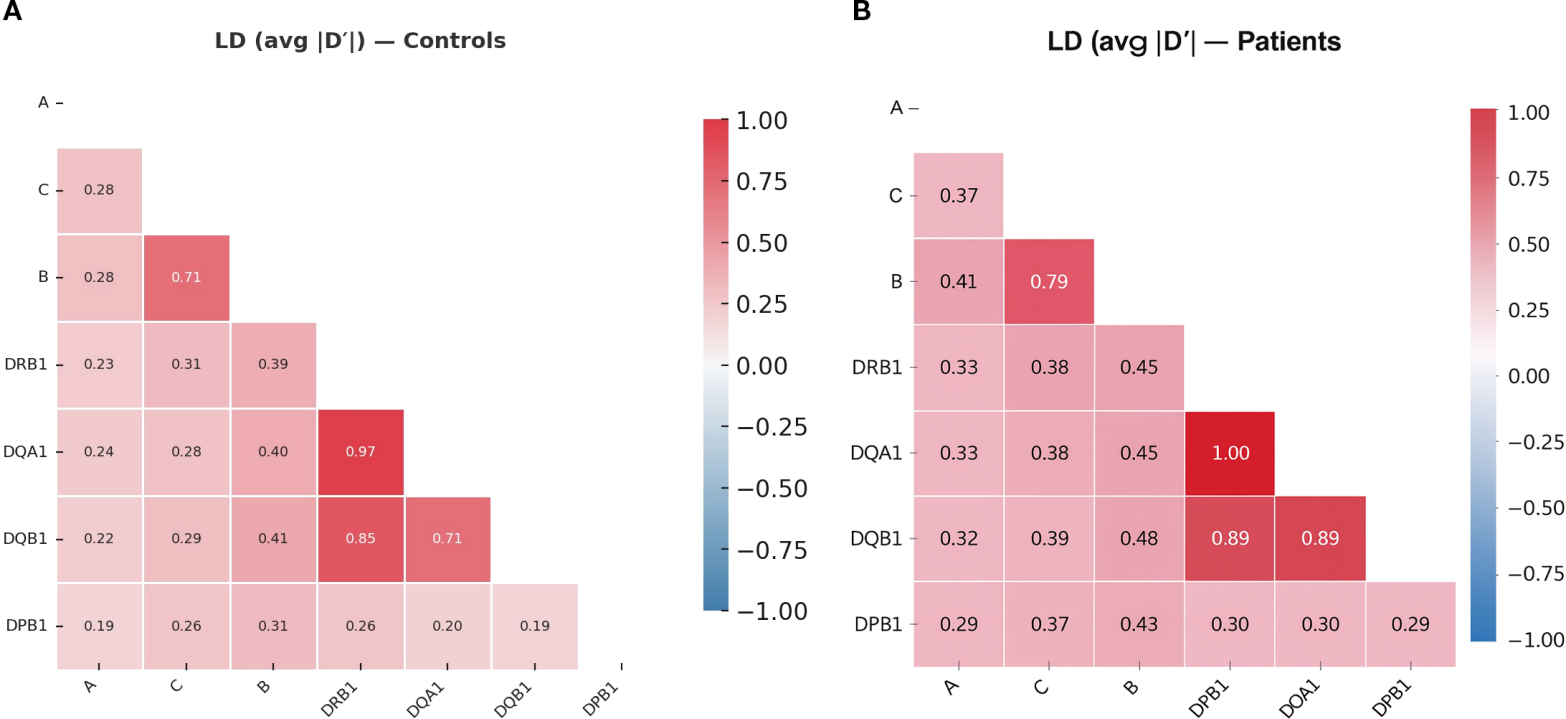
Linkage disequilibrium (LD) heatmap of HLA loci in aplastic anemia patients **(A)** and controls **(B)**. Pairwise LD between *HLA-A*, -*C*, -*B*, -*DRB1*, -*DQA1*, -*DQB1*, and -*DPB1* loci is shown as the mean absolute value of D′, estimated using the expectation–maximization algorithm from unphased genotypes. The heatmap’s color gradient, from white (weak) to dark blue (strong), reflects the degree of allelic co-segregation between locus pairs. Axis labels indicate HLA loci in chromosomal order.

Linkage disequilibrium (LD) analysis across *HLA-A*, *-C*, *-B*, *-DRB1*, *-DQA1*, *-DQB1*, and *-DPB1* loci revealed distinct haplotypic patterns, with stronger inter-locus LD observed in AA patients (**Figure 1A**) compared to healthy controls (**Figure 1B**). The most pronounced LD among AA patients occurred within the Class II region (*DRB1˜DQA1*, *DQA1˜DQB1*, and *DRB1˜DQB1*), consistent with their physical proximity, while moderate LD was noted between Class I and II loci (**Figure 1A**). In contrast, lower LD were seen in controls (**Figure 1B**), reflecting greater haplotypic diversity, suggesting that AA susceptibility is linked to the tighter, selective inheritance of immunogenetically relevant haplotypes involving Class II loci.

Accordingly, analysis was restricted to HLA Class II four-locus (*DRB1∼DQA1∼DQB1∼DPB1*) haplotypes and is presented in **Supplementary Table S2**. The initial negative association of *07:01:01˜01:02:01˜02:01:01˜04:01:01, 01:01:01˜01:01:01˜05:01:01˜04:01:01, 03:01:01˜02:01:01˜02:01:01˜04:01:01* (all at *p* = .027), as well as *15:01:01˜01:02:01˜06:02:01˜04:01:01, 04:01:01˜03:01:01˜03:01:01˜02:01:01, 04:01:01˜03:01:01˜03:01:01˜04:01:01* (all at *p = .032*) with reduced risk of AA was lost after applying the Bonferroni correction. The absence of Bonferroni-significant haplotypes despite strong Class II linkage disequilibrium reflects methodological limitations of EM-based haplotype inference under sparse data conditions. Notably, no specific two- or three-locus haplotypes were significantly associated with disease risk. The complete absence of several high-risk alleles from controls resulted in zero-frequency cells that destabilize maximum-likelihood haplotype estimation and inflate uncertainty around haplotype probabilities. This implies that susceptibility may be driven by individual allelic effects rather than extended haplotypic configurations (**Supplementary Table S3**).

### 3.4 Summary of key findings

This study revealed exceptionally strong associations between HLA class II loci, notably *DQA1* and *DPB1*, and AA. Four *DQA1* alleles, comprising DQA1*05:05:01, DQA1*03:03:01, DQA1*03:02:01, and DQA1*01:04:01, were found exclusively in AA patients, absent from all 250 controls, and showed very high adjusted ORs (69.368 to >100), all with corrected *p* < 0.0001. Similarly, DPB1*104:01:01 (OR = 111.584) and DPB1*02:01:02 (OR = 51.595) were strongly associated with increased AA risk. These unprecedented effect sizes, exceeding those typically reported in other AA cohorts (OR = 2–5) (15, 18, 19, 27–29), suggest these alleles may be key immunogenetic drivers of AA in the Kazakhstani population.

## 4 Discussion

Using high-resolution NGS-based HLA typing, this study identified exceptionally strong and population-specific associations between HLA Class II alleles and aplastic anemia in ethnic Kazakhs. In contrast to the modest and non-robust Class I signals, multiple *DRB1*, *DQA1*, *DQB1*, and *DPB1* alleles showed highly significant and biologically plausible associations with disease susceptibility, underscoring a dominant role for Class II–restricted immune mechanisms in AA pathogenesis in this population. This aligns with the immunopathological basis of AA as an autoimmune-mediated bone marrow failure, highlighted by the CD4^+^ T-cell dysregulation and aberrant antigen presentation (1, 2). These findings represent the first detailed HLA risk profile for a Central Asian population, highlighting that unique admixed ancestry requires tailored risk assessment tools and personalized treatment approaches (24). While select *DRB1*, *DQA1*, *DQB1*, and *DPB1* alleles were underrepresented among patients, suggesting protective roles, caution is warranted given the modest sample size and the emphasis on identifying risk-conferring alleles.

Several at-risk HLA-DRB1* alleles, including DRB1*05:05:01, DRB1*01:02:01, and DRB1*03:03:01, were significantly associated with a higher risk of AA in the Kazakhstani population. These associations were in apparent disagreement with an earlier Japanese study that reported DRB1*0405 as strongly positively associated with AA (27) and with studies on Chinese (18), Malaysian (28), and Korean (29) populations, which identified DRB1*1501 as an AA-susceptible allele. DRB1*07:01:01, DRB1*15:01:01, DRB1*04:01:01, and DRB1*05:03:01 were underrepresented in cases, and thus presumably protective of AA. This aligns with findings in Caucasian and some Asian populations that identified DRB1*0401, DRB1*0701, and DRB1*1501 as linked to a negative association (protective) with AA (19, 29). This population-specific association between DRB1 alleles and AA highlights how specific HLA alleles can confer susceptibility or protection against AA, depending on ethnic background.

The most significant finding of our study was the association between multiple class II *DQA1 and DPB1* alleles and susceptibility to AA. Notably, the high odds ratios for DQA1*05:05:01 and DQA1*03:03:01, and DPB1*02:01:02 and DPB1*104:01:01, coupled with the complete absence of certain *DQA1* and *DPB1* alleles in the patient group, stand out, suggesting that *DQA1* and *DPB1* variants play a crucial role in AA development. This contrasts with earlier studies on European (20) and East Asian populations (15, 19, 27, 29), which identified *DRB1* and *DQB1* as AA-associated loci. However, these studies did not include *DQA1* genotyping, underscoring the novelty of our findings. This indicates that the *DQA1* and *DPB1* loci, along with *DRB1* and *DQB1*, are key genetic factors influencing AA risk in the Kazakhstani population, likely by affecting autoantigen presentation and T-cell-mediated immune responses targeting hematopoietic stem cells (30).

The exceptionally strong association of DQA1*05:05:01, DQA1*03:03:01, DQA1*03:02:01, and DQA1*01:04:01 with AA (OR >69) indicates immunogenetic and population-structural factors. The absence of high−risk alleles such as DQA1*05:05:01 in controls reflects true population−restricted genetic structure rather than sampling bias. Kazakh populations are characterized by strong sub−ethnic and tribal stratification shaped by Turkic, Mongolic, and Persian ancestries, leading to uneven distribution of rare HLA haplotypes. Their enrichment in AA patients is consistent with immunogenetic founder effects and selective vulnerability rather than control misclassification. *DQA1* encodes the DQ heterodimer α−chain, which forms a high-affinity peptide−binding groove with DQB1 β−chains for hematopoietic self−antigens (31). Structurally, polymorphic residues within this α−chain shape peptide−binding repertoire, groove electrostatics, and complex stability, with substitutions at positions 52 and 75 stabilizing negatively charged pockets that bind basic, stress−induced self−peptides (32). This promotes the presentation of cryptic/damage−associated peptides from hematopoietic progenitors, lowers the CD4^+^ T−cell activation threshold, and drives immune−mediated marrow destruction characteristic of AA (32). Although the absence of these DQA1 alleles in controls inflates ORs, their recurrence across unrelated Kazakhstani patients supports a true biological effect, indicating that these variants act as potent AA susceptibility alleles rather than statistical artifacts (9, 11, 33).

The very large odds ratios for specific *DQA1*, *DQB1*, and *DPB1* alleles result from their complete absence in controls, rather than technical or population−genetic bias. All Class II loci are in HWE in controls, excluding genotyping errors, allele dropout, or hidden substructure. In rare−allele settings, zero−cell counts naturally yield large Fisher−exact ORs with wide CIs, indicating true enrichment rather than statistical inflation. ASR (Haberman) analysis, appropriate for sparse HLA tables, further supports this: alleles absent in controls but recurrent in cases show the highest residuals and are the main contributors to the χ² signal. Together, these results indicate that the extreme ORs represent genuine, highly penetrant population−specific susceptibility alleles, not statistical artifacts.

This is the first report to identify an association between *DQA1* alleles, specifically DQA1*05:05:01, DQA1*03:03:01, DQA1*03:02:01, and DQA1*01:04:01, found exclusively in patients, with adjusted odds ratios exceeding 69. We also identified *DQA1* alleles associated with reduced susceptibility to AA, namely DQA1*03:01:01, DQA1*01:01:01, and DQA1*05:01:01. Technical limitations of older typing methods (serological and low-resolution PCR) prevented routine examination of *DQA1*, so the study was primarily focused on *DRB1* and *DQB1*. The strong associations observed here with *DQA1* were a direct result of using NGS-based high-resolution typing (34). The high-resolution, 6-digit allelic resolution provided by NGS enabled the identification of significant *DQA1* variants and other rare variants, which were not feasible with conventional typing (34).

While not fully examined here, the strong population-specific links between HLA−DQA1 alleles and AA indicate that the DQA1 risk and protective variants could improve genetic risk stratification for individuals with idiopathic cytopenias or other haemoglobinopathies. We support the idea that high−resolution DQA1 typing may also enhance stem−cell donor selection, possibly by identifying immunogenetic profiles linked to disease susceptibility or immune−mediated marrow injury. Considering the role of HLA Class II molecules in antigen presentation and T−cell activation, DQA1 polymorphisms might ultimately aid in personalized treatment strategies, including predicting responses to immunosuppression. This warrants validation through larger prospective clinical studies.

A notable ethnic variation in HLA association patterns with altered AA susceptibility was documented across different populations. An earlier Japanese study identified DRB1*15:01-DQB1*06:02 as the primary risk haplotype for AA (27). A more recent, larger Japanese study confirmed associations between DRB1*15:01 and DQB1*06:02 and AA (15). However, these studies did not include *DQA1* in their analyses (15, 27) and thus could not assess its potential association (15, 27). Similarly, while DRB1*07:01 was found to be protective against AA among Koreans, no *DQA1* alleles were identified as linked to AA (29). It is tempting to speculate that these population-specific HLA patterns reflect adaptations to distinct pathogens and environmental stressors, suggesting that the pathways underlying AA may vary by ethnicity, challenging the idea of a single model for AA and other autoimmune bone marrow failure disorders (33).

A distinctive pattern emerged at the *DPB1* locus, in which DPB1*02:01:02 and DPB1*104:01:01 conferred increased risk, whereas DPB1*02:01:01 was protective, underscoring the value of NGS-based typing for distinguishing closely related alleles that would be indistinguishable using conventional 2− or 4−digit methods (34). Such opposing allele effects were rarely documented. Few studies have assessed population-specific DPB1 associations in AA, and none have identified DPB1*02:01:02 or DPB1*104:01:01 as risk alleles, emphasizing the novelty of these results. A European GWAS implicated Val76−encoding DPB1*03:01, DPB1*10:01, and DPB1*01:01 in severe AA (35), whereas an earlier European study found no association (20), and two Chinese studies similarly reported no significant DPB1 effects (18, 19). This suggests that *DPB1* associations may be ancestry−dependent, as seen in admixed Kazakhstani populations (9, 36), highlighting the need for large, ethnically diverse cohorts (37).

Compared with the strong Class II associations, Class I signals in Kazakhs were weak, with only B*40:02:01 showing increased AA risk. This aligns with Chinese (19) and Japanese (38, 39) reports implicating B*40:02, but contrasts with findings in North Americans (40) and multi−ethnic cohorts (16, 22, 37, 38) identifying B*14:02 as a risk allele, as well as studies in North Americans/Europeans linking B*07:02 to elevated AA risk (38). The initial protective trend of C*07:06:01 in Kazakhs differed from that in other populations, where C*05:01 increases risk (41), C*07:01 is protective in Indians (14), and C*04:01 predicts a better immunosuppressive therapy response in Chinese patients (17). In contrast to an Egyptian study reporting a strong association with A*02:01 (22), no *HLA−A* allele was linked to AA in Kazakhs, highlighting the need for ancestry−specific studies to refine genetic risk assessment and improve diagnostic precision.

Select *DRB1∼DQA1∼DQB1∼DPB1* haplotypes, notably those containing the DPB1*04:01:01 allele, showed notable but non-significant association trends. However, no specific two-locus or three-locus Class II or Class I (*A∼C∼B*) haplotypes reached statistical significance, likely due to diverse allelic expression (42). This lack of statistically significant haplotypes after multiple-testing correction should not be interpreted as evidence against a haplotypic contribution to AA risk, as it reflects insufficient statistical power and numerical instability of EM-based haplotype inference when rare or zero-frequency alleles are present (43). Our results are reminiscent of European reports linking the DRB1*03:01∼DQB1*02:01 haplotype to AA (20) but differed from Japanese findings implicating DRB1*15:01∼DQB1*06:02 as an at-risk haplotype (15) and Korean data showing protective effects of DRB1*07:01−containing haplotypes (29). European GWAS have also highlighted *HLA−DPB1* haplotypes with Val76−encoding alleles as risk factors for ALL (35). Several factors may explain these discrepancies: the very low frequency or absence of certain alleles in controls, which limits EM−based haplotype reliability; extensive admixture in Kazakhs, which likely weakens linkage signals (9, 24); and strong Class II interlocus LD, which can produce allelic redundancy when a single allele drives association. Together, these factors may mask multi−locus haplotype effects despite clear single−allele associations, reinforcing the need for population−specific genetic profiling in AA and related disorders.

In conclusion, this study identifies novel HLA Class II alleles—particularly DQA1 and DPB1—as key genetic determinants of AA susceptibility in the Kazakhstani population. Beyond their mechanistic relevance (11), these variants offer translational value for genetic risk stratification and refined donor matching (23). While population-wide screening remains impractical given AA’s rarity and limited rural access to HLA typing, targeted testing may be appropriate for individuals with familial marrow failure, unexplained cytopenias, or those undergoing donor evaluation. Incorporating high−resolution HLA data into donor registries could strengthen pre−transplant risk assessment (31, 34). As larger studies validate these associations, allele−based screening may support personalized surveillance and prevention strategies in high−risk Central Asian populations.

Our study has several strengths, notably the use of high-resolution NGS typing, which enabled us to achieve greater allelic precision than earlier studies and thereby to discover new associations, particularly at *DQA1*. NGS-based high-resolution typing was critical because it enabled the identification of associations with the *DQA1* locus and subtle allelic variants (34). By analyzing both Class I and Class II alleles within the same cohort, this study provides a comprehensive comparison of HLA-disease associations with AA in a Central Asian population. The modest AA sample size, typical of rare−disease studies, is balanced by a larger ethnically matched control group (n = 250). Zero−frequency alleles are expected in highly polymorphic HLA loci and do not bias association tests when appropriate sparse−data methods are applied. All Class II loci conformed to HWE in controls, excluding artefactual causes of zero counts. ASR results showed that these alleles contribute meaningfully to the χ² signal, supporting true biological enrichment. The close concordance between control allele frequencies and published Kazakh population data further validates the control cohort and argues against population stratification or recruitment bias as explanations for the observed associations.

However, the study has limitations that must be acknowledged. There was a lack of clinical outcome data, including information on disease severity, treatment modalities, response to immunosuppressive therapy, transfusion dependence, progression to severe AA, clonal evolution, relapse rates, or survival, thereby prompting the speculation whether the identified risk and protective alleles have prognostic value or predict treatment outcomes. Although the AA cohort (n = 91) is comparable to or larger than many prior HLA association studies, larger samples are needed to improve statistical power and detect weaker allelic or haplotypic effects for expanded analyses of genotype-phenotype patterns, disease severity, treatment response, and survival. Multi–center and registry-based studies across Kazakhstan and neighboring Central Asian populations are warranted to validate and verify and extend these findings. Other shortcomings include the strong linkage disequilibrium, which reduced Class I haplotype resolution and complicated the analysis of extended HLA effects. At the class II level, haplotype inference was constrained by sparse data and zero-frequency alleles, thereby reducing the reliability of EM-based haplotype estimation. This likely limited our ability to detect significant multi-locus Class II haplotypes despite strong linkage disequilibrium and robust single-allele associations (8). Furthermore, environmental, viral, and treatment-related factors were not assessed, and our study focused exclusively on ethnic Kazakhs. It did not include stratified analyses by ethnicity, despite Kazakhstan’s diverse population, raising the question of whether the observed associations are generalizable or population specific.

Despite these limitations, this study provides the first comprehensive HLA profile of AA in Central Asia, revealing new associations with *DQA1* and *DPB1* that lay a critical foundation for precision medicine in this genetically diverse yet understudied population. Replication studies in larger Kazakhstani and Central Asian cohorts are necessary to confirm these findings and determine their clinical relevance. Future work should include stratified analyses across Kazakhstan’s major ethnic groups to define ethnicity−specific risk profiles and support more personalized risk assessment and therapy. Functional studies of peptide binding, T−cell repertoires, and immune−signaling pathways linked to AA−associated DQA1 variants are now needed to validate these mechanisms and translate genetic associations into clinical practice.

## Data Availability

The datasets presented in this study can be found in online repositories. The names of the repository/repositories and accession number(s) can be found below: https://doi.org/10.17632/rwc2ryp5ks.1.
