# Supplementary material for: NGS identifies novel HLA-DQA1 and DPB1 associations with aplastic anemia in the Kazakhstani population
Source: Front Immunol. 2026 Feb 5;17:1752687. doi: 10.3389/fimmu.2026.1752687 (PMC12916586; doi:10.3389/fimmu.2026.1752687)
Supplement: Supplementary file 2 [file Table2.docx]

**Supplementary Table 2**

**HLA Class II Haplotypes in Patients and Controls**

| ***DRB1~DQA1~DQB1~DPB1* Haplotype** | **Total ^1^** | **Patients ^1^** | **Controls ^1^** | ***p*_adj_ ^2^** | **OR_adj_ ^2^ (95% CI)** | **Corrected *p* ^3^** |
| --- | --- | --- | --- | --- | --- | --- |
| *07:01:01~01:02:01~02:01:01~04:01:01* | 0.01023 | 0.00000 | 0.01286 | .027 | 0.202 (0.012–3.481) | .151 |
| *01:01:01~01:01:01~05:01:01~04:01:01* | 0.01023 | 0.00000 | 0.01286 | .027 | 0.202 (0.012–3.481) | .151 |
| *03:01:01~02:01:01~02:01:01~04:01:01* | 0.01023 | 0.00000 | 0.01286 | .027 | 0.202 (0.012–3.481) | .151 |
| *15:01:01~01:02:01~06:02:01~04:01:01* | 0.00909 | 0.00000 | 0.01143 | .032 | 0.226 (0.013–3.929) | .177 |
| *04:01:01~03:01:01~03:01:01~02:01:01* | 0.00909 | 0.00000 | 0.01143 | .032 | 0.226 (0.013–3.929) | .177 |
| *04:01:01~03:01:01~03:01:01~04:01:01* | 0.00909 | 0.00000 | 0.01143 | .032 | 0.226 (0.013–3.929) | .177 |

1. Haplotype frequencies
2. Adjusted for age and gender
3. Calculated according to Bonferroni correction: *pc* = 1 – [(1 – *p*)^n^]; n = number of comparisons
